# Supplementary figures and images for: Genetically dissecting the electron transport chain of a soil bacterium reveals a generalizable mechanism for biological phenazine-1-carboxylic acid oxidation
Source: PLoS Genet. 2024 May 6;20(5):e1011064. doi: 10.1371/journal.pgen.1011064 (PMC11108179; doi:10.1371/journal.pgen.1011064)

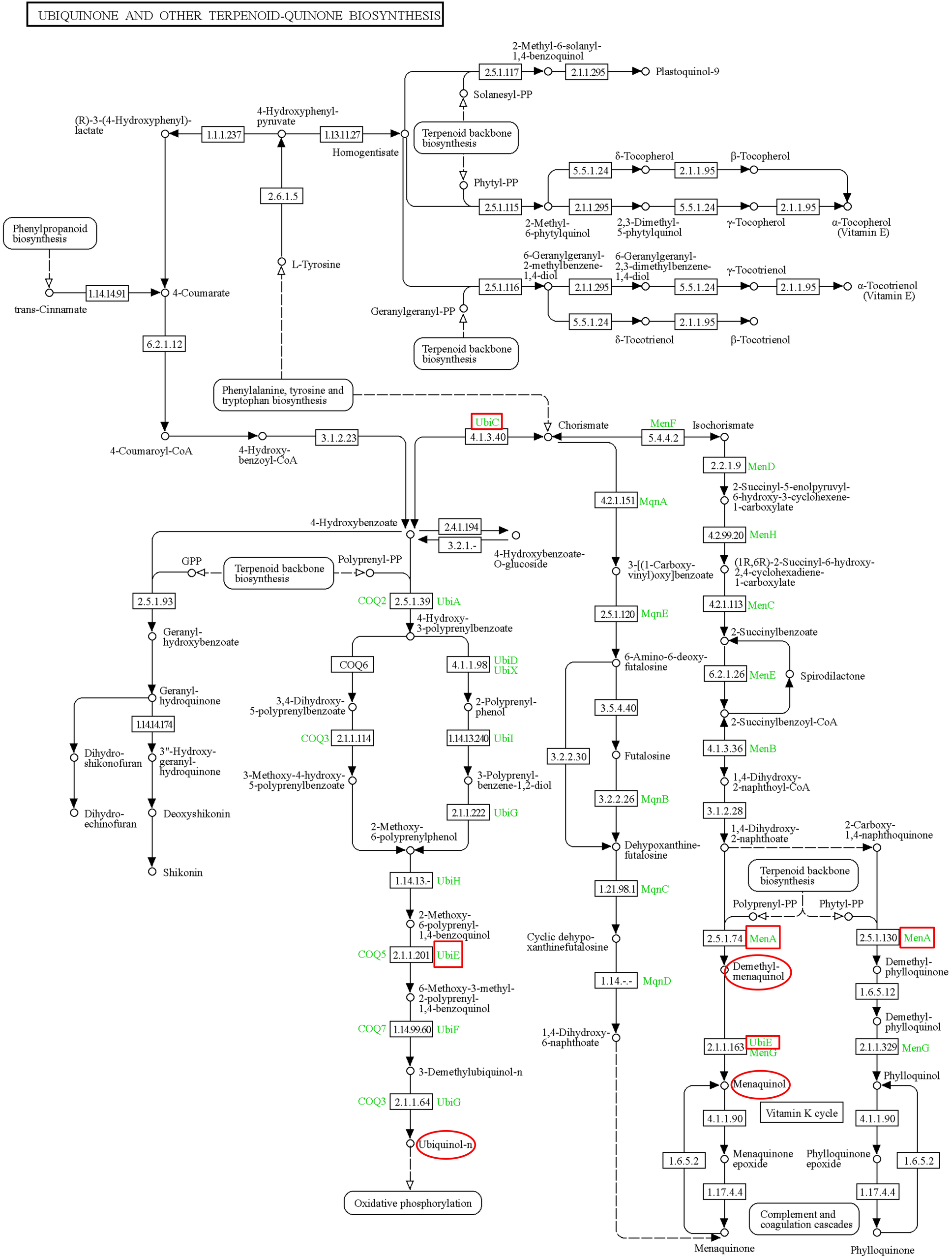

Supplement: S1 Fig — The relevant genes are indicated by red boxes and the relevant quinones by red ellipses. Note: MenG is a homolog to UbiE from photosynthetic organisms and is not present in γ-Proteobacteria like C. portucalensis MBL; the alternative pathway for menaquinone biosynthesis via futalosine (the Mqn genes) is also absent in C. portucalensis MBL [42–44]. Loss of UbiC results in the loss of ubiquinones. Loss of MenA results in the loss of menaquinones and demethylmenaquinones. Loss of UbiE results in the loss of ubiquinones and demethylmenaquinones. Note: Pseudomonas aeruginosa only has ubiquinones in its ETC under both aerobic and anaerobic growth conditions [31]. Pathway diagram used with permission from Kanehisa Laboratories (permission received November 6, 2023)[45]. (TIF) [file pgen.1011064.s001.tif]

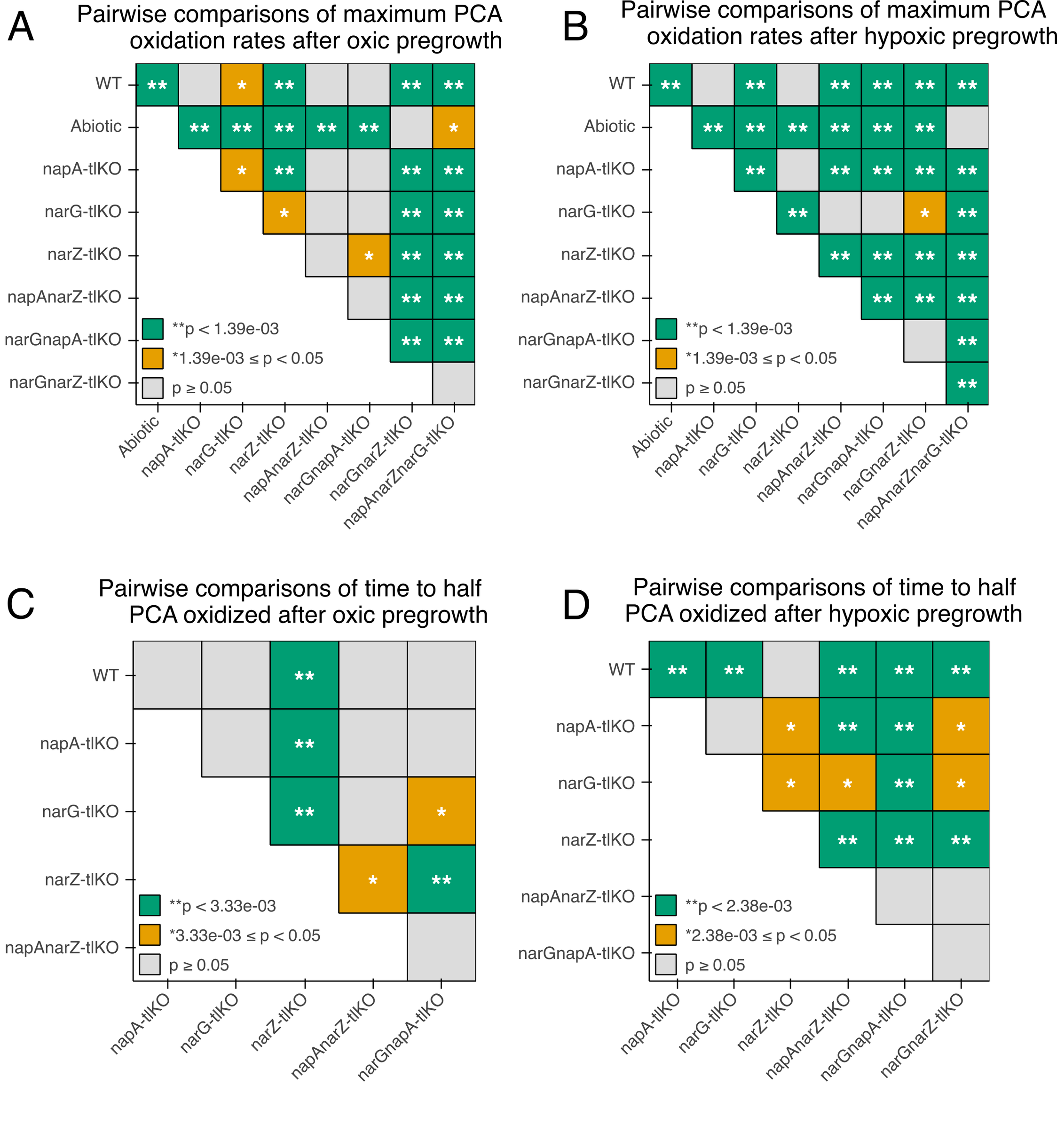

Supplement: S2 Fig — (A) Pairwise comparisons of the maximum PCA oxidation rate by all nitrate reductase genotypes after oxic pregrowth. This corresponds to Fig 3D. (B) Pairwise comparisons of the maximum PCA oxidation rate by all nitrate reductase genotypes after hypoxic pregrowth, corresponding to Fig 3E. (C) All pairwise comparisons for the time to oxidize half of the provided PCA, corresponding to Fig 3F. (D) Finally, all the pairwise comparisons for the half-max oxidation time for Fig 3G. (TIF) [file pgen.1011064.s002.tif]

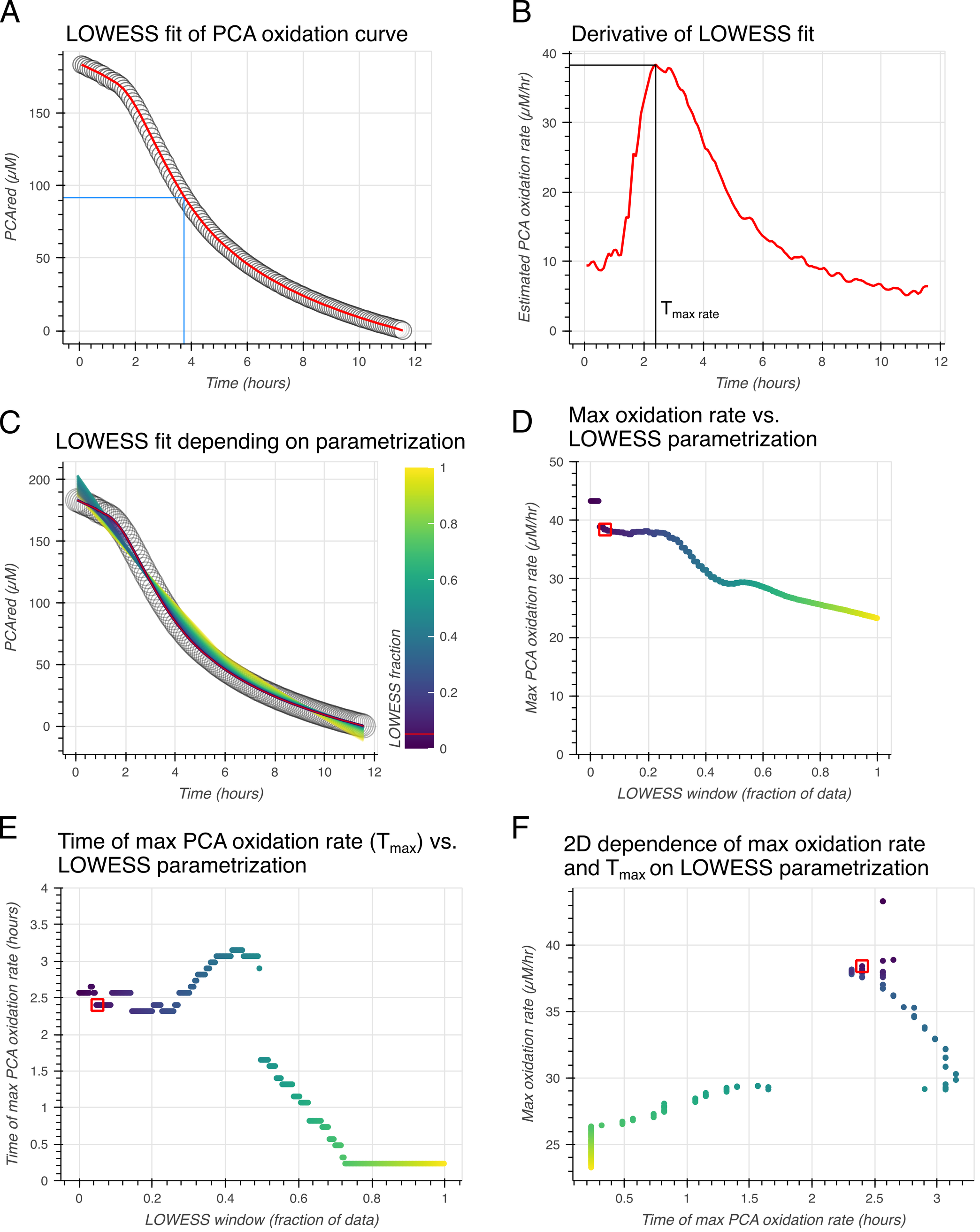

Supplement: S3 Fig — (A-B) A demonstration of the analysis pipeline. (A) We used a locally weighted scatterplot smoothing (LOWESS) algorithm to fit a curve to our empirical data, here showing an example of one of the biological replicates for wildtype C. portucalensis MBL oxidizing PCA with nitrate after hypoxic overnight pregrowth. The data are in semitransparent circles and the LOWESS fit is the red line. The fitting was parametrized to use 5% of the data in its sliding window. This fit allowed us to determine the time it took the cultures to oxidize half of the provided PCA (Thalf max, or any arbitrary threshold) and the derivative, or rate, of PCA oxidation. (B) This derivative allowed us to estimate the maximum rate of PCA oxidation and the time at which it occurred. The output of the LOWESS algorithm depends on one key parameter: the fraction of data that it considers for each smoothing window. (C) Examples of different fits to the same data as in Fig 4A, scanning the fraction parameter from 0 to 1. The value that was used for the analysis, 0.05, is in red. (D) How the estimated maximum PCA oxidation rate depends on the scanning window. The red square indicates the value at 0.05. (E) How the time to half of PCA being oxidized depends on the scanning window. The red square indicates the value at 0.05. (F) A 2D representation of both the maximum oxidation rate and the time at which it occurs. The red square indicates the value at 0.05. 0.05 was chosen as the window for all the analyses in this report as it appeared to give stable outputs while using a minimal window for fitting. (TIF) [file pgen.1011064.s003.tif]

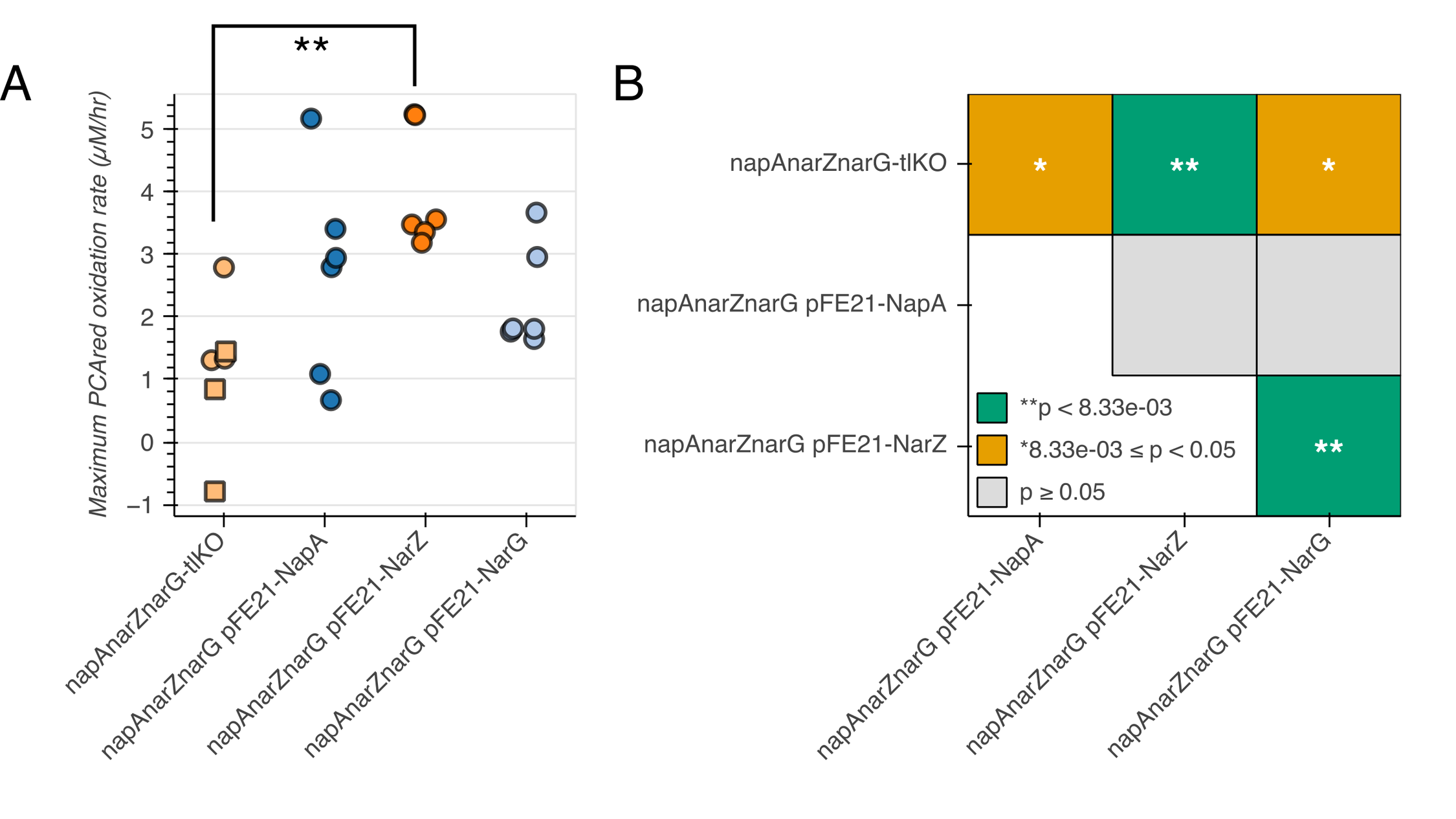

Supplement: S4 Fig — (A) The maximum PCA oxidation rate for the triple knockout strain and overexpressed individual nitrate reductases in that genetic background. Squares represent the means of technical triplicates and circles represent independent biological replicates. The negative value in the triple knockout background indicates that the cells were further reducing the provided stock of PCA, rather than oxidizing it. (B) Pairwise statistical tests against the null hypothesis that there is no difference between the mean maximum oxidation rates of two given genotypes. Given six comparisons, the Bonferroni-corrected p-value threshold for significance is p < 0.00833. (TIF) [file pgen.1011064.s004.tif]

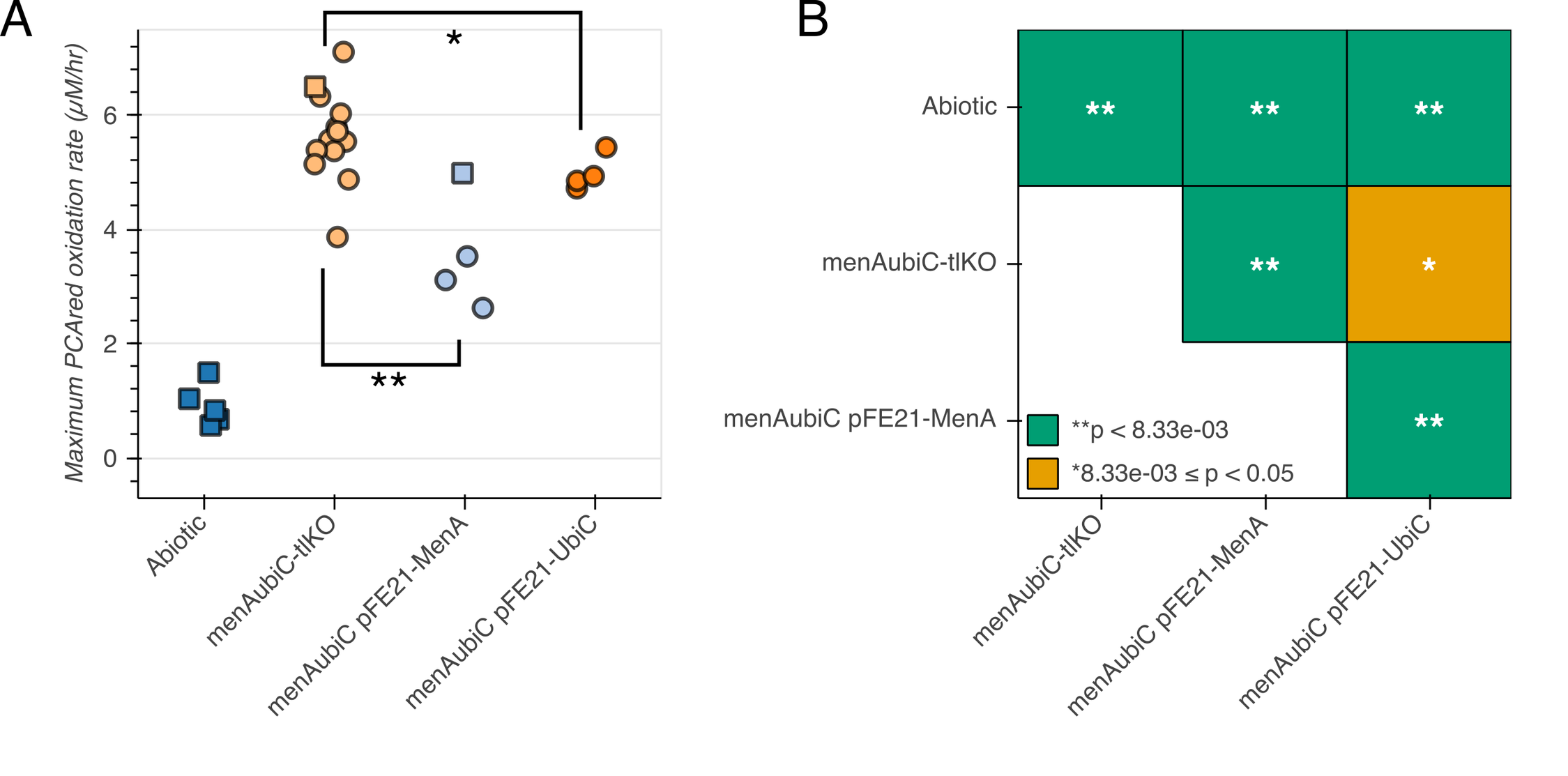

Supplement: S5 Fig — (A) Maximum PCA oxidation rates in complemented quinone knockout backgrounds. (B) Pairwise comparisons of the mean maximum PCA oxidation rates in (C). Given six comparisons, the Bonferroni-corrected threshold for significance is p < 0.00833. (TIF) [file pgen.1011064.s005.tif]

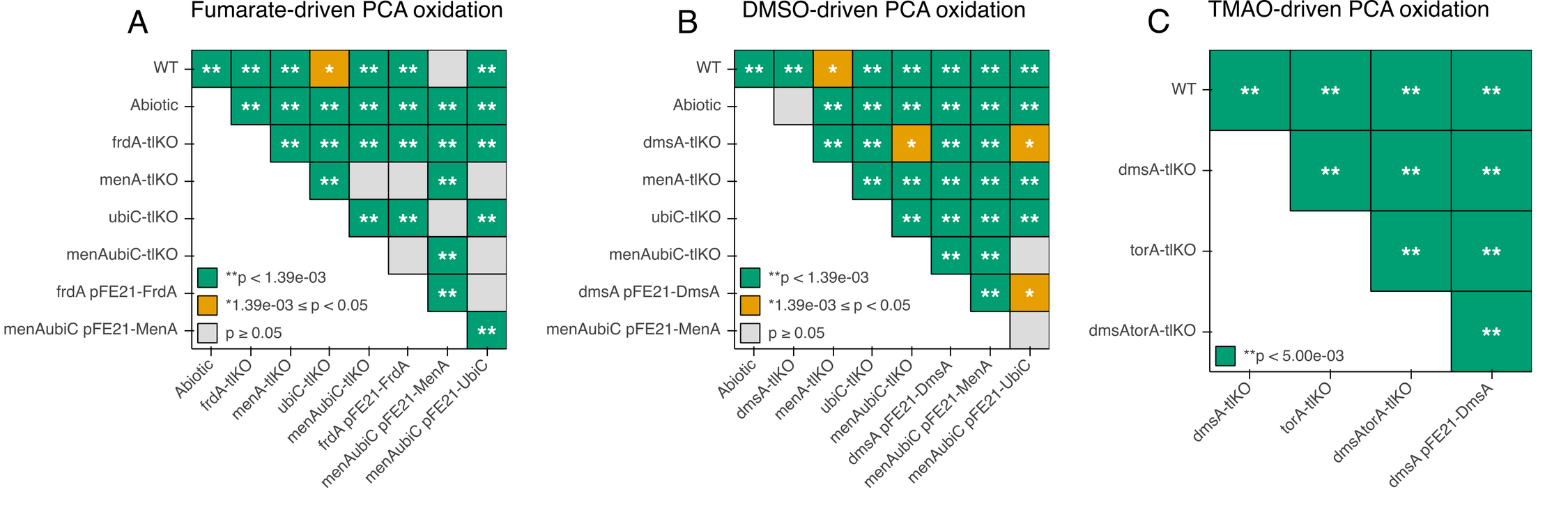

Supplement: S6 Fig — (A) Statistical significance matrix for the maximum PCA oxidation rate in the presence of fumarate. (B) Statistical significance matrix for the maximum PCA oxidation rate in the presence of DMSO. (C) Statistical significance matrix for the maximum PCA oxidation rate in the presence of TMAO. (TIF) [file pgen.1011064.s006.tif]

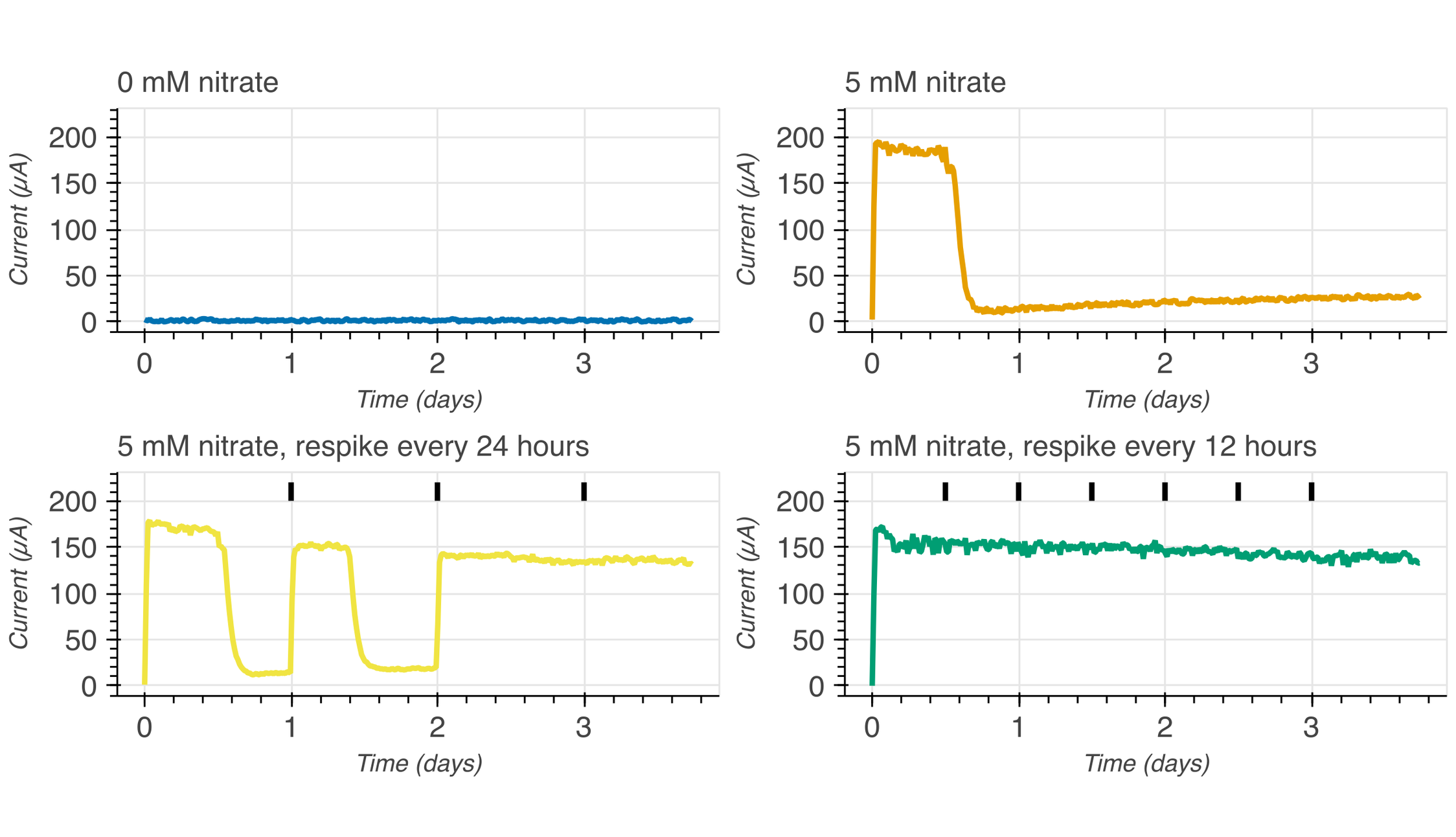

Supplement: S7 Fig — Each chart presents a time course of current (μA on a linear scale) during incubation of C. portucalensis MBL in a bioelectrochemical reactor with a working electrode that continuously reduces PCA. Current indicates that the culture is oxidizing PCA. Vertical black bars represent the timing of nitrate spiking, when appropriate. Each chart is titled according to the initial concentration of nitrate in the medium and the spiking schedule. (TIF) [file pgen.1011064.s007.tif]
