# Supplementary material for: Genetically dissecting the electron transport chain of a soil bacterium reveals a generalizable mechanism for biological phenazine-1-carboxylic acid oxidation
Source: PLoS Genet. 2024 May 6;20(5):e1011064. doi: 10.1371/journal.pgen.1011064 (PMC11108179; doi:10.1371/journal.pgen.1011064)
Supplement: S1 Text — (DOCX) [file pgen.1011064.s008.docx]

**Supplement**

***Supplementary results***

*A theoretical framework for understanding PCA oxidation metabolisms.*

In principle, many electron acceptors, both organic and inorganic, have the potential to oxidize PCA. Having previously found PCA oxidation to be coupled to nitrate reduction, we began our search for a general mechanism for PCA oxidation by performing a theoretical analysis of the reaction’s thermodynamics (Fig 1B). We considered the theoretical limit to the potential energy that could be conserved when PCA is oxidized by typical terminal electron acceptors used during anaerobic respiration. The thermodynamic favorability of these reactions is determined by their Gibbs free energy (∆G). The ∆G of a given reaction (∆G_r_) is related to the ∆G under standard conditions at pH 7 (∆Gº’_r_):

1. ∆G_r_ = ∆Gº’_r_ + RT ln(Q_r_)

Q_r_ is the quotient of the activities of the products and reactants, T is the temperature in Kelvin, and R is the ideal gas constant (8.314 J K^-1^ mol ^-1^). ∆Gº’_r_ can be derived from the midpoint potentials of reduction-oxidation (redox) reactions:

1. ∆Gº’_r_ = -nF∆Eº’_r_

Here, n is the number of electrons exchanged, F is the Faraday constant (96,485 J mol^-1^ V ^-1^), and ∆Eº’_r_ is the difference in the standard potential of the reactants. In the case of nitrate and phenazine-1-carboxylic acid (PCA), the analysis gives the following result:

1. PCA_oxidized_ + 2e^-^ + 2 H^+^ → PCA_reduced_ Eº’_1/2_ = -0.116 V;
2. NO_3_^-^ + 2e^-^ + 2 H^+^ → NO_2_^-^ + H_2_O Eº’_1/2_ = +0.433 V;
3. PCA_reduced_ + NO_3_^-^ → PCA_oxidized_ + NO_2_^-^ + H_2_O ∆Eº’_r_ = +0.549 V;
4. ∆Gº’_r_ = -2 × 96,485 × 0.549 J mol^-1^ = -106 kJ mol^-1^

Thus, under standard conditions at pH 7, the coupling of PCA oxidation to nitrate reduction gives a negative Gibbs free energy, meaning that it would be thermodynamically favorable. However, while we can control the pressure, temperature, and pH under laboratory conditions, there is no reason for the concentration conditions to be standard, and so it is important to consider at which ratio Q_r_ the reaction will become *unfavorable*. This can be derived as follows:

1. ∆Gº’_r_ + RT ln(Q_r_) ≥ 0;
2. ln(Q_r_) ≥ -∆Gº’_r_ / RT;
3. ln(Q_r_) ≥ 106 × 10^3^ / (8.314 × 298);

(10) Q_r_ ≥ 3.72 × 10^18^.

The coupling of PCA oxidation to nitrate reduction becomes unfavorable when the ratio of the activities of the product and reactant is over 18 orders of magnitude. In effect, this reaction will be favorable until it reaches completion, which matches our observation with *C. portucalensis* MBL, our model organism for studying PCA oxidation [1,2].

The same theoretical analysis applies to the other alternative terminal electron acceptors: fumarate (Eº’_1/2_ = +0.033 V), DMSO (Eº’_1/2_ = +0.160 V), TMAO (Eº’_1/2_ = +0.130 V), and nitrite (Eº’_1/2_ = +0.350 V) (Supplementary Table 1) [3,4]. During respiration, electrons travel from cytoplasmic reducing equivalents, such as NADH (Eº’_1/2_ = -0.320 V) [3], to the terminal electron acceptors via flux through the quinone/quinol pool in the cytoplasmic membrane. Conceivably, during their biologically catalyzed oxidation, phenazines may also donate their electrons to quinones. *C. portucalensis* MBL has the genes to synthesize three quinones: ubiquinone (UQ; Eº’_1/2_ = +0.113 V), menaquinone (MQ; Eº’_1/2_ = -0.074 V), and demethylmenaquinone (DMQ; Eº’_1/2_ = +0.040 V) (Fig 2 and S1 Fig). Based on their respective midpoint potentials, each of these would be expected to accept electrons from PCA (Fig 1B and S1 Table) [3,4]. Thus, it is feasible for bacteria to use these reactions during their metabolism.

***S1 Table. Thermodynamics of PCA oxidation coupled to anaerobic terminal electron acceptor reduction.***

| Electron acceptor | ∆Gº’_r_ (kJ mol^-1^) | Unfavorable boundary |
| --- | --- | --- |
| Nitrate | -106 | Q_r_ ≥ 3.72 × 10^18^ |
| Nitrite | -89.9 | Q_r_ ≥ 5.79 × 10^15^ |
| DMSO | -53.3 | Q_r_ ≥ 2.17 × 10^9^ |
| TMAO | -47.5 | Q_r_ ≥ 2.09 × 10^8^ |
| Ubiquinone (UQ) | -44.2 | Q_r_ ≥ 5.57 × 10^7^ |
| Demethylmenaquinone (DMQ) | -30.1 | Q_r_ ≥ 1.89 × 10^5^ |
| Fumarate | -28.8 | Q_r_ ≥ 1.10 × 10^5^ |
| Menaquinone (MQ) | -8.10 | Q_r_ ≥ 26.3 |

Different terminal reductases take electrons from different quinones, as determined by thermodynamic and kinetic constraints. For instance, nitrate has a sufficiently positive midpoint potential to favorably oxidize any of the quinones, while fumarate can only drive menaquinone oxidation to completion (Fig 1B). These redox relationships enable a conceptual model for interpreting PCA oxidation phenotypes: For instance, if ubiquinone is necessary for PCA oxidation, fumarate would not stimulate the process because its midpoint potential is such that it cannot oxidize ubiquinol (Fig 1B). The fact that PCA is abiotically oxidized by certain compounds leads to the hypothesis that biological PCA oxidation does not require specialized enzymes. To test this, we can distinguish whether PCA donates electrons to the terminal reductases via the quinol pool and, if it does, which of the quinones are at play. The hypothesis that quinones mediate phenazine oxidation in the bacterial electron transport chain is supported be the observation that ubiquinone-1 rapidly oxidizes the phenazines pyocyanin (PYO), phenazine carboxamide (PCN), and PCA in aqueous solution [5].

***Supplementary methods***

*Statistical analyses*

Given data that are not necessarily normally distributed, we employed bootstrapping for all our hypothesis testing, which we implemented as a two-tailed variant of the empirical t-statistic described by Efron and Tibshirani [6] (Algorithm 16.2 in the book). Specifically, when comparing two sets of datapoints, we tested the null hypothesis that they possessed the same means. We did this via the following algorithm:

1. Suppose you have two data sets X = {x_1_, x_2_, …, x_n_} and Y = {y_1_, y_2_, …, y_m_}.
2. If both sets have at least three elements, calculate the observed difference between their means:
   1. µ_x_ = 1/n Σ(X)
   2. µ_y_ = 1/m Σ(Y)
   3. d_obs_ = µ_x_ - µ_y_.
3. The observed t-statistic, t_obs_ = d_obs_ / √(σ_X_^2^/n + σ_Y_^2^/m), where σ_X_^2^ is the variance of X and σ_Y_^2^ is the variance of Y.
4. In order to generate the distribution that simulates the null hypothesis, shift the means of both sets to be equal:
   1. µ_tot_ = (Σ(X) + Σ(Y)) / (n + m)
   2. X’ = X - µ_x_ + µ_tot_
   3. Y’ = Y - µ_y_ + µ_tot_.
5. Generate the null hypothesis distribution by drawing 1,000,000 bootstrap replicates with replacement from the shifted data sets. For each replicate
   1. Draw a size-n sample with replacement from X’: x’
   2. Draw a size-m sample with replacement from Y’: y’
   3. d_b_ = 1/n Σ(x’) – 1/m Σ(y’)
   4. t_b_ = d_b_ / √(σ_x’_^2^/n + σ_y’_^2^/m), where σ_x’_^2^ is the variance of x’ and σ_y’_^2^ is the variance of y’.
6. Calculate the p-value of t_obs_ relative to the distribution of T = {t_1_, t_2_, … t_1,000,000_}:
   1. Count z, the number of elements of D, where |t_j_| ≥ |t_obs_| (the absolute values are what make this a two-tailed test)
   2. p = z/1,000,000.
7. For determining significance, apply the Bonferroni correction by dividing the standard threshold p-value (p = 0.05) by the number of concurrent pairwise comparisons. E.g., if there are 10 strains compared pairwise, statistical significance is at p < 0.005.

*Electrode chamber assay for PCA oxidation*

The electrode chamber assays were performed as described in Suzanne Kern’s PhD thesis [7], Chapter 7, with the following modifications:

1. The medium for the assay was the phenazine oxidizer basal medium, as described in the main text.
2. The glass components of the electrode chambers were extensively cleaned prior to each experiment: they were soaked in methanol for at least 2 hours, soaked in a potassium hydroxide solution overnight (added a couple drops of potassium hydroxide to chambers filled with lab-pure water), soaked in 10% hydrochloric acid overnight, and then muffled at 980 ºF for four hours, prior to assembly with stirbars and o-rings and autoclaving.
3. The polarity of the working and counter electrodes was flipped, with the working electrode poised to -500 mV relative to the reference electrode.
4. The graphite working electrodes (Alfa Aezar Cat. No. 14738) were polished with a Kim Wipe until shiny prior to soaking in 70% ethanol to sterilize them prior to chamber assembly.
5. The custom platinum mesh counter electrodes were also soaked in 70% ethanol to be sterilized prior to chamber assembly.
6. The reference electrodes were BASi Cat. No. MW-2030 instead of BASi Cat. No. RE-5B.
7. Instead of directly submerging the reference electrodes into the electrode chamber, they were inserted into ultrapure agarose (1% agarose in 3 M NaCl) in a 200 µL gel-loading pipette tip to create a stable salt bridge (also dipped into 70% ethanol to sterilize prior to use). The electrode-pipette tip connection was secured with Parafilm after the agarose had cured.
8. *C. portucalensis* MBL was pre-grown for these experiments as follows:
   1. Grow overnight 5 mL LB cultures at 30 ºC, shaking slanted tubes at 250 rpm.
   2. Measure OD_600_ of overnight cultures and inoculate 250 mL LB in a 1 L Erlenmeyer flask to an OD_600_ = ~0.06.
   3. Grow the 250 mL LB cultures, shaking at 250 rpm at 30 ºC, until they reach OC_600_ = ~2.8 (approximately 4-6 hours).
   4. Pellet all 250 mL in one bottle by spinning for 10 minutes at 6000 × g at room temperature.
   5. Resuspend cell pellet in 25 mL of the medium used for electrode chamber experiments and transfer to 50 mL conical tube.
   6. Twice more, pellet the cells by spinning for 8 minutes at 5500 × g at room temperature.
   7. After the second spin, resuspend in 6 mL of the experiment medium
   8. Measure OD_600_ and adjust to a final value of OD_600_ = 75.
   9. Bring cell suspension into mBraun anoxic chamber
   10. Inoculate 1 mL into each appropriate electrode chamber.
9. ATP and CFUs were measured as described in Glasser et al. [8]

1. Tsypin LM, Saunders SH, Bar-On Y, Leadbetter JR, Newman DK. Draft genome sequence of the redox-active enteric bacterium *Citrobacter portucalensis* strain MBL. Microbiol Resour Announc. 2020 Aug 6;9(32):e00695-20.

2. Tsypin LM, Newman DK. Nitrate reduction stimulates and is stimulated by phenazine-1-carboxylic acid oxidation by *Citrobacter portucalensis* MBL. mBio. 2021 Aug 31;12(4):e02265-21.

3. Thauer RK, Jungermann K, Decker K. Energy conservation in chemotrophic anaerobic bacteria. Bacteriol Rev. 1977 Mar;41(1):100–80.

4. Unden G, Bongaerts J. Alternative respiratory pathways of *Escherichia coli*: energetics and transcriptional regulation in response to electron acceptors. Biochimica et Biophysica Acta (BBA) - Bioenergetics. 1997 Jul;1320(3):217–34.

5. Ciemniecki JA, Newman DK. NADH dehydrogenases are the predominant phenazine reductases in the electron transport chain of *Pseudomonas aeruginosa*. Molecular Microbiology. 2023 Feb 24;00:1–14.

6. Efron B, Tibshirani R. An introduction to the bootstrap. Nachdr. Boca Raton, Fla.: Chapman & Hall; 1998. 436 p. (Monographs on statistics and applied probability).

7. Kern SE. Consequences of redox-active phenazines on the physiology of the opportunistic pathogen *Pseudomonas aeruginosa* [Internet]. Massachusetts Institute of Technology; 2013. Available from: https://dspace.mit.edu/handle/1721.1/80985

8. Glasser NR, Kern SE, Newman DK. Phenazine redox cycling enhances anaerobic survival in *Pseudomonas aeruginosa* by facilitating generation of ATP and a proton-motive force: Phenazines facilitate energy generation. Molecular Microbiology. 2014 Apr;92(2):399–412.

9. Basset GJ, Latimer S, Fatihi A, Soubeyrand E, Block A. Phylloquinone (Vitamin K1): Occurrence, Biosynthesis and Functions. Mini-Reviews in Medicinal Chemistry. 2017;17(12):1028–38.

10. Hiratsuka T, Furihata K, Ishikawa J, Yamashita H, Itoh N, Seto H, Dairi T. An Alternative Menaquinone Biosynthetic Pathway Operating in Microorganisms. Science. 2008 Sep 19;321(5896):1670–3.

11. Vo CDT, Michaud J, Elsen S, Faivre B, Bouveret E, Barras F, Fontecave M, Pierrel F, Lombard M, Pelosi L. The O2-independent pathway of ubiquinone biosynthesis is essential for denitrification in *Pseudomonas aeruginosa*. Journal of Biological Chemistry. 2020 Jul 3;295(27):9021–32.

12. Kanehisa M, Goto S. KEGG: kyoto encyclopedia of genes and genomes. Nucleic Acids Res. 2000 Jan 1;28(1):27–30.

**Supplementary Figure Captions**

***S1 Fig. KEGG reference pathway for quinone biosynthesis (map00130).*** The relevant genes are indicated by red boxes and the relevant quinones by red ellipses. Note: *MenG* is a homolog to *UbiE* from photosynthetic organisms and is not present in γ-Proteobacteria like *C. portucalensis* MBL; the alternative pathway for menaquinone biosynthesis via futalosine (the *Mqn* genes) is also absent in *C. portucalensis* MBL [1,9,10]. Loss of *UbiC* results in the loss of ubiquinones. Loss of *MenA* results in the loss of menaquinones and demethylmenaquinones. Loss of *UbiE* results in the loss of ubiquinones and demethylmenaquinones. Note: *Pseudomonas aeruginosa* only has ubiquinones in its ETC under both aerobic and anaerobic growth conditions [11]. Pathway diagram used with permission from Kanehisa Laboratories (permission received November 6, 2023) [12].

***S2 Fig. All pairwise comparisons of PCA oxidation dynamics of nitrate reductase knockouts, including shaking (oxic) versus standing (hypoxic) overnight pregrowth.*** (A) Pairwise comparisons of the maximum PCA oxidation rate by all nitrate reductase genotypes after oxic pregrowth. This corresponds to Fig 3D. (B) Pairwise comparisons of the maximum PCA oxidation rate by all nitrate reductase genotypes after hypoxic pregrowth, corresponding to Fig 3E. (C) All pairwise comparisons for the time to oxidize half of the provided PCA, corresponding to Fig 3F. (D) Finally, all the pairwise comparisons for the half-max oxidation time for Fig 3G.

***S3 Fig. Quality of LOWESS fit depends on parametrization of its scanning window.*** (A-B) A demonstration of the analysis pipeline. (A) We used a locally weighted scatterplot smoothing (LOWESS) algorithm to fit a curve to our empirical data, here showing an example of one of the biological replicates for wildtype *C. portucalensis* MBL oxidizing PCA with nitrate after hypoxic overnight pregrowth. The data are in semitransparent circles and the LOWESS fit is the red line. The fitting was parametrized to use 5% of the data in its sliding window. This fit allowed us to determine the time it took the cultures to oxidize half of the provided PCA (T_half max_, or any arbitrary threshold) and the derivative, or rate, of PCA oxidation. (B) This derivative allowed us to estimate the maximum rate of PCA oxidation and the time at which it occurred. The output of the LOWESS algorithm depends on one key parameter: the fraction of data that it considers for each smoothing window. (C) Examples of different fits to the same data as in Figure 4A, scanning the fraction parameter from 0 to 1. The value that was used for the analysis, 0.05, is in red. (D) How the estimated maximum PCA oxidation rate depends on the scanning window. The red square indicates the value at 0.05. (E) How the time to half of PCA being oxidized depends on the scanning window. The red square indicates the value at 0.05. (F) A 2D representation of both the maximum oxidation rate and the time at which it occurs. The red square indicates the value at 0.05. 0.05 was chosen as the window for all the analyses in this report as it appeared to give stable outputs while using a minimal window for fitting.

***S4 Fig. Complementation of nitrate reductase knockouts after overnight standing pre-growth.*** (A) The maximum PCA oxidation rate for the triple knockout strain and overexpressed individual nitrate reductases in that genetic background. Squares represent the means of technical triplicates and circles represent independent biological replicates. The negative value in the triple knockout background indicates that the cells were further reducing the provided stock of PCA, rather than oxidizing it. (B) Pairwise statistical tests against the null hypothesis that there is no difference between the mean maximum oxidation rates of two given genotypes. Given six comparisons, the Bonferroni-corrected p-value threshold for significance is p < 0.00833.

***S5 Fig. Overexpression of MenA and UbiC does not complement the PCA oxidation phenotype of the* menAubiC *double mutant.*** (A) Maximum PCA oxidation rates in complemented quinone knockout backgrounds. (B) Pairwise comparisons of the mean maximum PCA oxidation rates in (C). Given six comparisons, the Bonferroni-corrected threshold for significance is p < 0.00833.

S***6 Fig. All pairwise comparisons for the strains in Figure 6.*** (A) Statistical significance matrix for the maximum PCA oxidation rate in the presence of fumarate. (B) Statistical significance matrix for the maximum PCA oxidation rate in the presence of DMSO. (C) Statistical significance matrix for the maximum PCA oxidation rate in the presence of TMAO.

***S7 Fig. PCA oxidation depends on nitrate availability in a bioelectrochemical reactor.*** Each chart presents a time course of current (µA on a linear scale) during incubation of *C. portucalensis* MBL in a bioelectrochemical reactor with a working electrode that continuously reduces PCA. Current indicates that the culture is oxidizing PCA. Vertical black bars represent the timing of nitrate spiking, when appropriate. Each chart is titled according to the initial concentration of nitrate in the medium and the spiking schedule.
