# Supplementary material for: Genetically dissecting the electron transport chain of a soil bacterium reveals a generalizable mechanism for biological phenazine-1-carboxylic acid oxidation
Source: PLoS Genet. 2024 May 6;20(5):e1011064. doi: 10.1371/journal.pgen.1011064 (PMC11108179; doi:10.1371/journal.pgen.1011064)
Supplement: Attachment — Submitted filename: TsypinEtAl2024_response.pdf [file pgen.1011064.s010.pdf]

Dear Editor:

Thank you for your work in processing this paper and collecting the reviewers' feedback. Their comments are very constructive and have helped us think more deeply about our manuscript and to tailor it for our audience. Overall, it appears that Reviewers 1 and 2 asked us to clarify the text and data presentation, while Reviewer 3 was satisfied with the paper as submitted. We believe that our responses and edits to the text have addressed all of the reviewers' concerns, allowing the manuscript to meet the standard for publication. We have formatted the documents according to the submission guidelines for PLoS Genetics, with each figure in separate files (pre-processed by PACE), the figure captions at the end of the document, and with the supplementary materials in a separate file. We appreciate your consideration of our response below, where the Reviewers' remarks are in red, and our replies are in black:

Reviewer #1: In this paper, Tyspin et al., report studies to provide genetic evidence for biological oxidation of phenazine-1-carboxylaic acid (PCA) a compound with previously reported ability to support extracellular electron transfer in bacteria. It is well known that reduced PCA can participate in extracellular electron transfer, but the relevant abiotic and biological pathway(s) for its oxidation are largely unknown. In this work the authors perform experiments to analyze the relative contribution of abiotic PCA oxidation, the ability of PCA to reduce bacterial quinones to their respective quinols, or the ability of reduced PCA to donate electrons to one or more alternative respiratory chains that are often only present or more abundant under anaerobic conditions. This is an important and unresolved question in the field that often does not have well-controlled studies or makes conclusions without the needed supporting data.

For these above reasons, it is my opinion that the findings of this work should be of interest to many readers and could set the stage for many important subsequent studies. However, the current version of the manuscript lacks important information that will allow many readers to appreciate the results and allow others to repeat many of the experiments. Thus, it is my opinion that some major rewriting, revisions, deletions and additions to the paper are needed.

We thank Reviewer 1 for their comments and recommendations, and we appreciate their recognition that our work contributes to the field. There is significant overlap between the suggestions that the three reviewers have given us, so there are some instances where we address Reviewer 1's concerns by implementing feedback from Reviewer 2 or 3, which we indicate explicitly below.

1. The presentation of the work makes it difficult for the reader to follow and obtain the major conclusions of the work. For example, the focus on Category 1, 2 and 3 routes for PCA oxidation may be obvious to the authors but this reader found themselves constantly referring to the text to remember what each of these categories were. The text should simply state abiotic oxidation, PCA reduction of quinone, or transfer to an alternative terminal oxidase.

We have implemented this revision throughout the manuscript to increase the clarity and accessibility of the categories. In some instances, this amounted to reversing parenthetical

statements (putting “Category X” in the parenthetical and forefronting the explicit description). In others, we kept the context as it was (e.g., “the abiotic control rules out Category 3 PCA oxidation”). In yet others, we dropped the use of “Category” entirely. With all due respect to the reviewer, we find judicious usage of the Category terms to be clarifying, and hope our edits throughout the text have now made it easier to follow.

2. The Results section reads more like an introduction to the work or a Discussion of the data than a presentation of the experimental data. On one hand it takes almost 3 pages before an experiment is described and results presented. In addition, there is little if any reference to primary data in this section to guide the reader through the text and help them understand how the experimental data leads to conclusions.

We interpret the concern as referring to the theoretical treatment of PCA oxidation mechanisms. While we respectfully disagree that these are not primary results of our work, we are implementing the recommendation from Reviewer 2 and moving this section to the supplement to streamline the results section.

3. It is a concern that I can not find anything in the paper (M&M or supplementary) that reports the amount of alternative electron acceptor present in the media. Without this information, others will not be able to reproduce the experimental design or data in this paper.

This information is available on the protocols.io that is linked to in our methods, but we have also added an explicit line in the main text of the methods. (It is always 10 mM of the terminal electron acceptor for the plate reader assays.)

4. The data presentation also makes it hard for others to reproduce experiments in the future. There is little primary data (growth curves, primary data for rates of PCA oxidation, quinone reduction etc) presented for the wild type or mutant strains of both organisms. Instead relative data are presented with not information on actual data. Also in the studies with mutants, the reader does not know if the difference in the rate of PCA oxidation (by any route) reflects a difference in the growth of the cultures when a quinone biosynthetic or alternative electron transfer pathways if blocked.

We believe that there is a misunderstanding here: the cells are non-growing during the PCA oxidation assays because they are provided with only reduced PCA as an electron donor and nitrate, fumarate, DMSO, and TMAO as terminal electron acceptors. In our previous publication, (https://doi.org/10.1128/mbio.02265-21), Figures S2 and S3 demonstrate a lack of growth with nitrate and nitrite. While fumarate can function as both a carbon source and a terminal electron acceptor, it cannot function as both at the same time (the TCA cycle cannot proceed in opposite directions at once at the same node). Finally, all of the raw data, including OD600 measurements and analyses are accessible through GitHub and CaltechDATA repositories, which we believe to meet a high standard for reproducibility and interpretation.

5. The Discussion and interpretation of the data is often based on the “assumption” that the control of the alternative election acceptor enzymes in these organisms will be the same or at least very similar to that in E. coli. With the advent of genomics, it is clear that the oxygen regulation of alternative electron transfer pathways is often not the same. Thus, I would caution the authors about making conclusions in the Discussion without additional experiments, in subsequent papers.

We believe there is a philosophical disagreement here: the advent of genomics is what allows us to make this assumption. We have sequenced and published the genome of *C. portucalensis* MBL (https://doi.org/10.1128/mra.00695-20), and used the direct similarities that we observed to hypothesize about the regulation of PCA oxidation-relevant genes. Our results match those expectations and previously published work from other labs.

6. I may have mis-understood the logic (for reasons mentioned above), but I could make a case that PCA reduction of quinone is as important, or maybe even more important, that the use of PCA as an electron donor to one or more of the alternative oxidases based on the analysis of mutants in both species.

We try to make the argument that both are important. We have revised to make this more explicit in the discussion. Specifically, see the sentence: “Our observations that (i) any respirable terminal electron acceptor stimulates PCA oxidation and (ii) that the presence of quinones is required for a large fraction of the oxidation rate imply a generalizable mechanism: any cell that can perform anaerobic respiration ***and has electron flux through the quinol pool*** should oxidize PCA.”

7. In the case of PCA reduction of quinones, can a single electron transfer from reduced PCA can generate a semi-quinone. Semi-quinone formation could generate reactive intermediates and a stress response that could impact how one interprets the data.

Phenazine radicals from single electron transfers do form, but this is generally under acidic conditions and their presence is easy to detect because their color (both visible spectra and fluorescent properties) are very different in their fully reduced, fully oxidized, and radical forms. We didn’t observe the evolution of PCA radicals in our experiments. Most of the studies with phenazine radicals have focused on pyocyanin (the canonically most cytotoxic phenazine) and those studies have led to the following conclusions:

- Friedheim and Michaelis (1931, https://doi.org/10.1016/S0021-9258(18)76619-2) provide a very helpful phase diagram (Figure 7) that reports electrochemical pyocyanin radical formation below pH 5.
- O’Malley et al. (2004, https://doi.org/10.1152/ajplung.00025.2004) report that the pyocyanin radical can form by oxidizing glutathione under acidic conditions and by oxidizing NAD(P)H under neutral conditions. However, we note that this is not a direct comparison to our case, as in our study the reduced phenazine-1-carboxylic acid would need to be oxidized to the radical, rather than reduced to it.

- Kang, Cho, and Lee (2022, https://doi.org/10.1128/spectrum.02312-22) report that ferrous iron reduces pyocyanin and 1-hydroxyphenazine, but not phenazine-1-carboxylic acid, to their radicals in a one-electron transfer, leading to cytotoxicity.

This is all to say that we're not aware of any direct studies of PCA radical formation under physiologically-relevant conditions, but the available evidence and our colorimetric observations suggest that under our conditions PCA radicals are not dominant. As a thought experiment, if PCA radicals were forming under our conditions, it would not change our interpretation very much: In our previous publication (https://doi.org/10.1128/mbio.02265-21), we observed that PCA oxidation and nitrate reduction proceed stoichiometrically. This means that even if PCA were releasing one electron at a time, both of its available electrons eventually make their way to the terminal electron acceptor. Furthermore, our rate measurement for PCA oxidation tracks the loss of fluorescence, meaning that by the time the solution is non-fluorescent, all the PCA has been oxidized. So even if the oxidation is stepwise, the ultimate result and the final rate of complete oxidation remain consistent with our reported results. As far as stress response goes, the conditions of our experiments are definitely stressful to the cells, phenazine redox notwithstanding. It would require a significant effort to rigorously tease apart the effects and consequences of carbon source starvation, anaerobic respiration, and phenazine redox cycling, which is beyond the scope of this study.

8. Along these same lines, it is possible that electron transfer to nitrate can generate RNS that cause a stress response and complicate the interpretation of the data?

Studying nitrosyl radical formation would require an entire EPR study, which is beyond the scope of this report, but our response to whether this complicates the interpretation of our data is the same as above: even if those radicals were forming, our results from our prior work (https://doi.org/10.1128/mbio.02265-21) show that the end-state of PCA-mediated nitrate reduction is nitrite, and any intermediary radicals, while adding to the tapestry of the process, would not alter our main conclusions.

Reviewer #2: In this manuscript, Tsypin et al. dissect the molecular mechanisms underlying biological phenazine oxidation in Citrobacter portucalensis MBL and Pseudomonas aeruginosa. They develop new genetic tools for C. portucalensis MBL over the course of the study to also establish this strain as a new model to study extracellular electron transfer processes in microorganisms.

This is a careful, detailed and foundational study that establishes a genetic framework for biological phenazine oxidation and its physiological impact on microbes. In terms of presentation, the experimental design and the underlying rationale were conveyed beautifully and the data visualization is on point. I also want to commend the rigorous and thoughtful statistical analyses conducted throughout this study. My only criticism is that the paper is far too long and meandering (especially in the results section) and needs to be condensed substantially to make it punchier and easier to read. A few suggestions to condense the paper are listed below:

We thank Reviewer 2 for their encouraging feedback, concrete recommendations, and stimulating conceptual questions. Our detailed responses are below.

• Move the theoretical framework to the supplement (lines 131-208). While it is really important, and should be a part of this paper, the details are somewhat tangential. The authors can mention which reactions have a positive or negative ΔG in the main text and refer to the supplement for more details.

Done, thank you for the suggestion. While we are partial to Reviewer 3's take on this section, we recognize the consensus between you and Reviewer 1 and have moved this section to the supplement.

• Refrain from describing all pairwise comparisons for mutant strains. While I appreciate the comprehensive genetic analyses that is conducted in this study, I was starting to get a little lost in the weeds when all the mutants were compared to each other under a number of different conditions. It would be preferable to only describe the phenotypes of mutants that are important for the story and let the readers infer the rest from the figures. Less is more!

Thank you for pointing this out. We made efforts to clarify and streamline throughout the text, and adapted our figures according to your recommendations below.

• Move the description of the statistical analyses to the supplement. Again, a really important part of the paper but the details are tangential to the main storyline and, as a result, distracting. I would also recommend moving this section to the supplement and referring to it in the main text or the figure legends as appropriate.

Done. We appreciate your recognition that our description of the approach is important!

• Refrain from repeating results in the discussion. The discussion can be condensed to describe an overarching framework for PCA oxidation and its relevance in the context of the microbe and the environment as well as the limitations of the study. Details re: specific fellow up experiments are nice but not as useful.

Thank you for this suggestion. We have revised our discussion to make it clearer, but we also want to note the feedback from Reviewer 3, who thought it was well written. There is clearly a tension between preferred styles here, and we have attempted to edit the text accordingly.

Additional, more specific and minor comments are listed below:

• Lines 246-253 is more relevant for the methods section. Since one type of genetic perturbation system is used after an initial trial, it might only be worth bringing it up in the methods, just to streamline things a bit.

We revised this section to be more streamlined, but we kept the comparison between different knockouts because we think that the fact that homologous recombination and oligo-mediated recombination can be equivalent is an important result to communicate.

• Figure 3 and Figure 4 can be combined. I think Figure 4 panels A, B, E, and F can all be removed, and the rates in panels B and C can be combined with Figure 3. Additionally, I think the “time to half oxidation” plots are a very interesting piece of data that can be moved out of the Sup into main text. I would include Sup Fig 4.2 panels A and B in the new version of Figure 3 that has the time course experiments and the rate plots. From there, you can explain that only rates will be used in the next few figures. Also, figure 4 legend contains information that should be only in the main text. Keep analysis of the results in the text.

Thank you for this suggestion, which we have implemented.

• Figure 5 and the associated quinone analysis is very informative. However, I think the paper is lacking justification as to why a UbiE mutant was not made. This would be the most important mutant to make to separate out and rank which quinones contribute the most to the oxidation of PCA. This should be discussed more in the discussion.

While we agree making this mutant would have been ideal, LMZT discovered the *ubiE* gene right before he left the lab which is why he did not make it. Because he has moved on to his postdoc where he is focused on very different things, we must leave the construction of this mutant for another day. We have revised the text (line 362-366) to note that making this mutant is a priority for future research.

• Line 421 – “exacerbated” should be “marginally rescued” maybe?

We think “exacerbated” is correct here: a rescued phenotype would have a faster rate of PCA oxidation. The fact that the *menA* overexpressing strain has a lower PCA oxidation rate than the *menAubiC* double knockout indicates that the phenotype is more severe.

• Lines 446-447, I would emphasize the regulatory mechanisms of each nitrate reductase in P. aeruginosa are opposite in C. portucalensis. This will help drive home that the phenotypes make sense later when the regulatory pathways are discussed in more detail.

Thank you for this suggestion—implemented!

• Figure 6, can the abiotic control line also be included? Was one done in this experiment?

The abiotic control (reduced PCA and nitrate without cells) was done in the experiments shown in Figures 3 and 4, but not in the same 96-well plate as the *P. aeruginosa* mutant comparisons. However, we did perform this control with a phenazine-null strain of *P. aeruginosa* in our previous work (please see https://doi.org/10.1128/mbio.02265-21, Figure 1). It did not feel

appropriate to include the abiotic control from a previous experiment in the same figure, so we omitted it.

• Figure 7, remove panels C, F, I. Move stats to supp. Keep mutants in the same order across panels for ease of reading

Done! It's not possible to keep the mutants in quite the same order because there are different mutants for different terminal electron acceptors, but we've tried to keep it as consistent as possible.

• Line 681 – "Figure 12" is a typo

Thank you!

In terms of the scientific content, I have a few conceptual questions

1) The authors repeatedly mention that PCA oxidation might be beneficial under anaerobic conditions where electron donors are limited. I am confused by this statement as I would expect that fermentation, which is more common under anaerobic conditions, would lead to the excretion of reduced organic compounds that can readily serve as electron donors. If the authors can provide any evidence that electron donors are indeed limiting under anaerobic conditions or in anoxic environments that would be useful to bolster some of the central claims re: the relevance of PCA oxidation in this study.

You are correct that the environmental relevance of phenazine (and other redox-active metabolite) oxidation is speculative at this point. Clearly, follow-up studies are needed. However, to respectfully push back, we note: 1) In many ecological systems, there are multiple gradients of electron acceptors following the order of their relative redox potentials (e.g., oxygen to nitrate to ferric iron to sulfate), and there are many forms of respiration co-occurring in anoxic environments where electron donors may be limited and the rate of fermentation is relatively low. 2) There is a conceptual analogy between our observation, which could be called facilitated respiration, and the already recognized phenomenon of facilitated fermentation that occurs with phenazines and other redox-active metabolites. 3) One of the advantages of using reduced PCA to supplement the ETC, rather than a fermentation product, is that the PCA can donate electrons without needing active transport into the cytoplasm. Ultimately, it's hard to imagine that cells wouldn't be using all the tools available to them, especially given how ubiquitous and promiscuous molecules like PCA are. Thus we feel our speculation on this topic is appropriate.

2) I remain confused about the mechanistic details of PCA oxidation coupled to nitrate reduction based on the genetic evidence presented in this study. The authors clearly demonstrate that a triple KO of C. portucalensis lacking all three nitrate reductases is indistinguishable from the abiotic control (Figure 4C and 4D). The authors also show that a menAubiC KO can barely oxidize PCA (Figure 5A). These data, to me, are consistent with two alternate hypotheses:

• The nitrate reductase complex is responsible for oxidizing PCA and requires an input of electrons from the quinone pool to do so – not sure how and why?; in the absence of the nitrate reductase complex, the quinone pool can oxidize PCA minimally, likely because it is difficult for PCA (hydrophilic) to access quinones in the membrane
• The quinone pool oxidizes PCA and requires a functional nitrate reductase complex to regenerate itself; in the absence of quinones maybe the nitrate reductase complex has some residual capacity to oxidize PCA

I am not quite sure if it is possible to distinguish between these two hypotheses purely based on genetic data. My impression from reading the discussion is that the authors favor the latter hypothesis over the former but I am not quite sure what the underlying reason might be. It would be nice if, in the discussion, the authors state various plausible hypothesis that are consistent with their genetic data for PCA oxidation.

Thank you for these questions; we have revised the discussion to clarify our view on what is happening (see lns. 377-391). We agree that future biochemical experiments are needed to fully understand the mechanisms at play, but this is outside the scope of this study. For example, a key question is just how soluble PCA and other redox metabolites are in the membrane, and there is a PhD student in our lab who is systematically determining the phase partitioning coefficient for these compounds (for a more conceptual discussion, please see https://doi.org/10.1016/j.cbpa.2023.102320).

While we are not modifying the discussion to explain what follows (in the interests of space), here we provide some more information that is relevant and explains why we favor the second hypothesis. In the first alternative hypothesis above, you write, "in the absence of the nitrate reductase complex, the quinone pool can oxidize PCA minimally, likely because it is difficult for PCA (hydrophilic) to access quinones in the membrane." However, the condition when all terminal reductases are knocked out is equivalent to a condition when the terminal electron acceptor is not provided. Under these conditions, we actually observe that the cells *reduce* any available PCA (please see https://doi.org/10.1128/mbio.02265-21, Figure S1). Other recent work from the lab, albeit in a *P. aeruginosa* and not *C. portucalensis*, demonstrated that the NADH dehydrogenases are the dominant phenazine reductases in the cell, reducing the phenazines without a quinone intermediary (https://doi.org/10.1111/mmi.15049). While indirect, the above evidence suggests that there is no net rate of PCA oxidation by quinones when the nitrate reductase complexes are absent, and this net rate would be necessary for this alternative hypothesis to be viable. As you note, it is not at all clear how or why the nitrate reductase complex would require an input of electrons from the quinone pool in order to also take electrons from PCA.

Nonetheless, this can all be directly addressed by the following experiment: Severely starve the cells prior to the experiment (or use isolated membrane vesicles) such that there is no endogenous electron source. If these cells cannot oxidize PCA during anaerobic respiration despite having the full complement of quinones, then the process follows the first alternative

hypothesis you listed above. If the PCA oxidation proceeds as normal, this supports the second alternative hypothesis.

3) None of the experiments in this study address whether PCA oxidation coupled to nitrate/fumarate/DMSO/TMAO reduction facilitate growth or energy conservation in the organism. While the bioreactor experiment in figure 8 show that PCA oxidation enhances survival, the underlying basis is unclear. It would be useful if the authors could perhaps discuss how cells produce ATP/NADH during PCA oxidation coupled to nitrate reduction and whether C. portucalensis can couple this process with CO2 fixation or heterotrophic growth.

We have added concise commentary on this point to the discussion. Please see lns. 432-438.

4) For the bioreactor experiments, I feel like there is a control missing from this analysis with a reactor that contains PCA and nitrate, but no cells. With electrodes poised at such a low reduction potential, is there a chance that the electrode can catalyze the oxidation of PCA and reduction of nitrate in the absence of cells? This might be detectable as current but could also be measured as a readout of nitrite produced.

Thank you for this question. In our bioelectrochemical setup, it isn't possible to drive the reduction of nitrate and the oxidation of PCA at the same time abiotically. At worst, it would be conceivable for the working electrode to simultaneously reduce both PCA and nitrate, but this doesn't happen: Figure 8B shows that there is no appreciable current with only nitrate and the electrode, indicating that the electrode does not reduce nitrate to nitrite. This recent review underscores the need for new catalysts and electrodes to make the electrochemical reduction of nitrate to nitrite possible: https://doi.org/10.1039/D3TA01592K

Furthermore, our abiotic controls in Figures 3 and 4 show that nitrate doesn't oxidize PCA abiotically, and this remains the case in our bioelectrochemical chamber setup, which we confirmed, but did not explicitly discuss in our report: PCA was reduced by the electrode and the current arrested after total reduction (i.e., nitrate alone did not redox cycle PCA with the electrode prior to the experiment start). This is evident from the very beginning of the time course in Figure 8B: The current starts from zero at the time of inoculation, and the cells are inoculated into chambers that had had a poised electrode continuously reducing PCA in the presence of nitrate. The fact that the current was at zero to begin with indicates that there was no electron transfer from PCA to nitrate prior to the addition of the cells.

Reviewer #3: In this work, Tsypin et al. examine PCA oxidation as a driver of respiration in bacteria. A major finding is that PCA oxidation is common, and likely happens via a generalizable mechanism. This likely flow of electrons through the quinone pool to various terminal reductases. My overall reaction is very positive; I think there are some important findings here that will be of broad interest. In general, redox coupling, Gibbs free energy, etc. can make for complex reading, especially from a theoretical standpoint. However, the authors here have done a fantastic job of explaining these processes in clear language, ensuring that

novices and experts alike will find it accessible. This is critical for a paper like this, which I think will be well-received by a broad audience. In the discussion, the authors propose a logically satisfying model that closes the phenazine loop (Fig 9A) in the context of competition and mutualism.
This work is very strong, and the presentation is excellent. I only have the minor concerns below.

We really appreciate Reviewer 3's positive assessment of our work. It was a challenge to present the breadth of our work clearly, and it is encouraging that it came across in a compelling way. We took to heart the feedback from Reviewers 1 and 2 for making our narrative more concise, but we hope that the original spirit of our manuscript still comes through.

Minor concerns:

L85: "react quickly enough with phenazines to re-oxide them abiotically" Should it be 're-oxidize' instead of 're-oxide?

Thank you, fixed!

L170-171: Does the ETC involve cytochromes? Or is it simply NADH dehydrogenasequinone poolterminal reductase? It might be useful to provide map of the whole chain as you understand it in C. portucalensis MBL, as some readers may wonder if there are other ETC components that PCA might interact with.

As we understand it, the only cytochromes in the anaerobic ETC are in the periplasmic nitrate reductase (NapC is a cytochrome) and in the Nrf complex (a nitrite reductase that doesn't seem to be active in our experimental conditions, based on the fact that all of the nitrite evolved during nitrate respiration isn't lost. See LMZT's dissertation, page 41, Figure 18: doi.org/10.7907/rmsf-e465). In that regard, the only missing components in the ETC are the NADH dehydrogenases. After this publication, there is a more nuanced argument to be made: other work from our lab (https://doi.org/10.1111/mmi.15049) reports that NADH dehydrogenases are the dominant phenazine **reductases** in *P. aeruginosa*. This means that a complete treatment of the ETC would include the potential phenazine redox cycle that is driven by the activity of the NADH dehydrogenases and flux through the quinol pool. This is probably too complex for the scope of this report, but it is something that we plan to posit as a coherent picture in the future.

L384: It would be nice if this section ended with a concise statement of what the reader should take from these experiments. While I appreciate that this is noted in the next section, I would suggest that rounding out this section is important given the amount experiments presented.

Thank you for the suggestion. We added the sentence, "Summarily, PCA oxidation is abolished only when all three terminal nitrate reductases are knocked out, and any terminal nitrate

reductase can drive PCA oxidation when it is expressed in the pregrowth condition" to the end of this section.

L540: "cultures that received addition nitrate" should be "cultures that received additional nitrate"

Corrected, thank you!

L552: "Despite the PCA oxidation current ceasing between days one and three, the effect on survival was not apparent until day four, on which the cultures with PCA began to die (Figure 8C)." This is confusing to me. When I look at 8C, several of the grey lines/circles decline at day 4. The legend says that those represent the setup with no PCA. This seems at odds with the quoted statement. To me, it looks like the one with PCA shows less death than the one without it. Is this the basis for the claim that PCA oxidation is beneficial? If it is, it would be good to see a separate plot of the data on day 4, that clearly shows all the data points (maybe similar to Fig 7A, for example). Right now it is difficult to assess the difference.

You are absolutely right, this was a typo! The text now reads "on which the cultures **without** PCA began to die."

L605: It would be great to hear in this paragraph how the authors envision transfer of electrons from PCA to the quinone pool. Do they envision it happening directly, or no?

The short of it: we don't have any direct evidence for either mechanism, so it feels premature to speculate too concretely. However, recent work by another lab member (https://doi.org/10.1111/mmi.15049) demonstrates that, in aqueous solution, ubiquinone spontaneously and instantaneously oxidizes phenazine-1-carboxylic acid, phenazine-1-carboxamide, and pyocyanin. This means that the electron transfer can very well be direct. It's an open question whether this is the case when the cell membrane and protein complexes are at play. We added a succinct discussion of this point to the end of that paragraph, which now reads (lines 366-373):

"While the *ubiCmenA* double knockout had a more severe PCA oxidation phenotype than the menA knockout alone, the *ubiC* single knockout phenotypes and the complementation results suggest that (demethyl-)menaquinones play a more significant role than ubiquinones (e.g., Figures 5A and 8A). Recent work from our lab demonstrated that ubiquinone spontaneously and instantaneously oxidizes PCA in aqueous solution (31); our results presented here imply that the plasma membrane environment changes the dynamics of how phenazines and quinones interact. This motivates further dissection of the role of specific quinones during in vivo PCA oxidation in future studies."
