# Supplementary material for: Genetically dissecting the electron transport chain of a soil bacterium reveals a generalizable mechanism for biological phenazine-1-carboxylic acid oxidation
Source: PLoS Genet. 2024 May 6;20(5):e1011064. doi: 10.1371/journal.pgen.1011064 (PMC11108179; doi:10.1371/journal.pgen.1011064)
Supplement: Attachment — Submitted filename: Tsypin PLoS Genetics 2023.docx [file pgen.1011064.s009.docx]

In this paper, Tyspin et al., report studies to provide genetic evidence for biological oxidation of phenazine-1-carboxylaic acid (PCA) a compound with previously reported ability to support extracellular electron transfer in bacteria. It is well known that reduced PCA can participate in extracellular electron transfer, but the relevant abiotic and biological pathway(s) for its oxidation are largely unknown. In this work the authors perform experiments to analyze the relative contribution of abiotic PCA oxidation, the ability of PCA to reduce bacterial quinones to their respective quinols, or the ability of reduced PCA to donate electrons to one or more alternative respiratory chains that are often only present or more abundant under anaerobic conditions. This is an important and unresolved question in the field that often does not have well-controlled studies or makes conclusions without the needed supporting data.

For these above reasons, it is my opinion that the findings of this work should be of interest to many readers and could set the stage for many important subsequent studies. However, the current version of the manuscript lacks important information that will allow many readers to appreciate the results and allow others to repeat many of the experiments. Thus, it is my opinion that some major rewriting, revisions, deletions and additions to the paper are needed.

1. The presentation of the work makes it difficult for the reader to follow and obtain the major conclusions of the work. For example, the focus on Category 1, 2 and 3 routes for PCA oxidation may be obvious to the authors but this reader found themselves constantly referring to the text to remember what each of these categories were. The text should simply state abiotic oxidation, PCA reduction of quinone, or transfer to an alternative terminal oxidase.
2. The Results section reads more like an introduction to the work or a Discussion of the data than a presentation of the experimental data. On one hand it takes almost 3 pages before an experiment is described and results presented. In addition, there is little if any reference to primary data in this section to guide the reader through the text and help them understand how the experimental data leads to conclusions.
3. It is a concern that I can not find anything in the paper (M&M or supplementary) that reports the amount of alternative electron acceptor present in the media. Without this information, others will not be able to reproduce the experimental design or data in this paper.
4. The data presentation also makes it hard for others to reproduce experiments in the future. There is little primary data (growth curves, primary data for rates of PCA oxidation, quinone reduction etc) presented for the wild type or mutant strains of both organisms. Instead relative data are presented with not information on actual data. Also in the studies with mutants, the reader does not know if the difference in the rate of PCA oxidation (by any route) reflects a difference in the growth of the cultures when a quinone biosynthetic or alternative electron transfer pathways if blocked.
5. The Discussion and interpretation of the data is often based on the “assumption” that the control of the alternative election acceptor enzymes in these organisms will be the same or at least very similar to that in *E. coli*. With the advent of genomics, it is clear that the oxygen regulation of alternative electron transfer pathways is often not the same. Thus, I would caution the authors about making conclusions in the Discussion without additional experiments, in subsequent papers.
6. I may have mis-understood the logic (for reasons mentioned above), but I could make a case that PCA reduction of quinone is as important, or maybe even more important, that the use of PCA as an electron donor to one or more of the alternative oxidases based on the analysis of mutants in both species.
7. In the case of PCA reduction of quinones, can a single electron transfer from reduced PCA can generate a semi-quinone. Semi-quinone formation could generate reactive intermediates and a stress response that could impact how one interprets the data.
8. Along these same lines, it is possible that electron transfer to nitrate can generate RNS that case a stress response and complicate the interpretation of the data?
